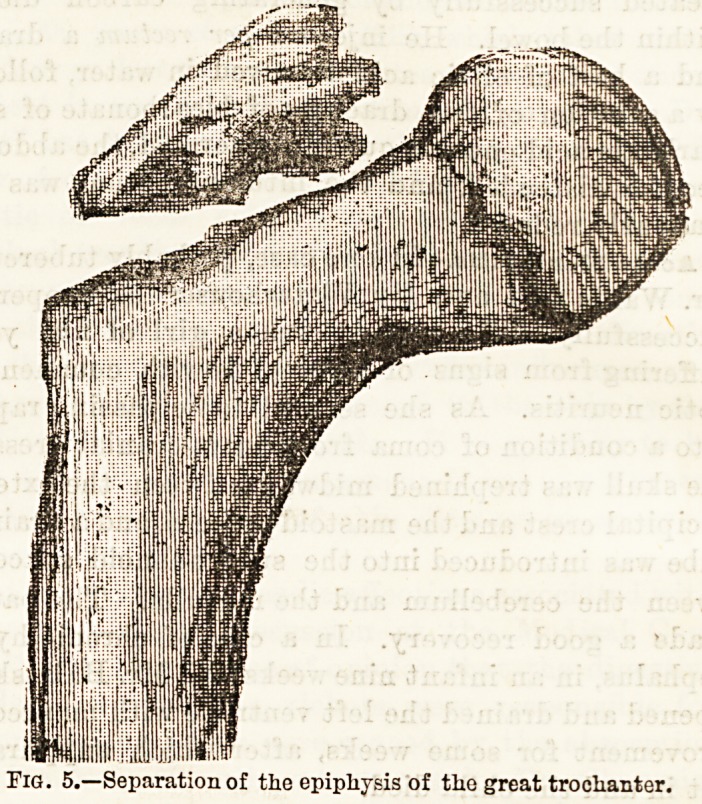# Bone, Joint, and Orthopædic Surgery

**Published:** 1894-08-04

**Authors:** 


### PROGRESS IN SURGERY.

### BONE, JOINT, AND ORTHOPÆDIC SURGERY.

### Dislocations.—*Continued.*

**Internal Disorganisation of the Knee-joint.**—C. B. Lockwood[9] detailed a case of dislocation of the internal semi-lunar fibro-cartilage, which was operated on, as the joint was apt to lock under circumstances which endangered the patient's life. The anterior third of the cartilage was found to have been torn from all its tibial attachments, and was therefore removed, the remainder being sewn down to the tibia. Twenty-one months after the accident the joint was perfect, but the patient feared to play tennis or football because of the danger of a sudden twist. H. Allingham, in the discussion, said that he had opened the knee-joint 35 times, and only once with a bad result, in the sense of having a stiff joint afterwards. Stanley Boyd[10] gave notes of a case of a man, aged 24, who had, while playing football three years ago, fallen with one of the players on him, and his legs crossed under him. Since then the joint had repeatedly given way, and he had recently wrenched it. At times it became locked in the semi-flexed position. There was great looseness of the capsule, and the tibia was not displaced backwards. Boyd considered it a case of rupture of the anterior crucial ligaments.

### Congenital Dislocations.

**Of the Hip.**—A. H. Tubby[11] points out that the term dislocation here is a misnomer since, in such instances, there is no real or perfect acetabulum from which the head of the femur can be dislocated. In truly congenital cases the capsular ligament is enormously thickened and elongated, and the iliac portion of the acetabulum is suppressed. The cavity is triangular and

Fig. 2.—Showing a separation of the external epicondyle. (Hamilton.)

Fig. 3.—Showing separation of the internal epicondyle.

not round. Hence it is impossible for the head of the femur to fit into it. The round ligament is as often present as absent. Unfortunately, recognition of the abnormal condition is not often made till the time when the child can walk. He advocates treatment by complete extension on the lines laid down by Buckminster Brown and William Adams, followed by the use of appropriate walking instruments and crutches. The object of this treatment is to obtain shortening of the elongated capsule, which, being already thickened, especially at its upper part, proves sufficiently strong to retain the head of the bone close to its natural position. Tubby is able to record two successful cases. Hoffa[12] gives the details of an autopsy on a child three years of age operated on by his method for double congenital dislocation, who died six months later from diphtheria. It was found that the result of the operation was the formation of a completely new articulation. The articular surfaces were covered everywhere by hyaline cartilage; the cotyloid cavity was deep, furnishing a good support for the head of the femur, and everywhere perfectly smooth, owing to the investing cartilage.

**Congenital Luxation of the Patella.**—Schon[13] reports a case of a girl, aged thirteen, with congenital dislocation of the left patella. The patient's mother and one sister (both deceased) had similar deformities of both knees. The patient suffered no inconvenience in walking, and the movements were perfect. The author has collected twenty-six cases of this deformity, of which three were hereditary.

### Fractures.

**Fractured Patella.**—Ballance[14] showed at the Medical Society of London two cases of fractured patella occurring in patients aged 36 and 45 years, who had been treated by immediate incision and wiring. The vertical incision which he used was placed laterally, so as to avoid a scar over the front of the patella. He had operated in seven cases, all of which were uniformly successful. All other forms of operation failed to remove the aponeurosis, which slipped between the bones and the clot which had accumulated there, and kept the fragments apart.

Fig. 4.—Separation of the lower epiphysis of the femur.

**Injuries to the Epiphyses and their Results.**—Jonathan Hutchinson, junior,[15] quotes several cases of separation of the epiphyses of the olecranon and two undoubted cases of the upper epiphyses of the radius. He also alludes to the peculiar deformity that occurs in separation of the lower epiphyses of the radius. A. H. Tubby,[16] writing on traumatic separation of the epiphyses of the slower extremity, gives a full account of the subject, with details of individual cases. The most important lesion is separation of the lower epiphyses of the femur (see Fig. 4). Thirty cases and specimens of this injury are recorded. In thirteen the history was very uniform, the leg having been twisted in a wheel. That definite diagnostic point, the forward displacement of the lower fragment even on to the front of the upper is strongly insisted upon as a means of distinction from fracture in that situation. In the latter event the lower fragment is displaced backwards. Of that rare injury, traumatic separation of the epiphyses of the great trochanter, six instances are given, three of which were verified by autopsy (see Fig. 5).

Fig. 5.—Separation of the epiphysis of the great trochanter.

[9] Lancet, March 17th, 1894, p. 673. [10] Lancet, March 3rd, 1894, p. 543. [11] The Hospital, May 12th, 1894. p. 119. [12] Med. Week, April 27th, 1894, p. 194. [13] Ep. Brit. Med. Jour., February 24th, 1894, p. 29. [14] Lancet, January 27th, 1894, p. 208. [15] Brit. Med. Jour., March 31st, 1894, p. 669. [16] Annals of Surg., March, 1894.

## Figures and Tables

**Fig. 2. f1:**
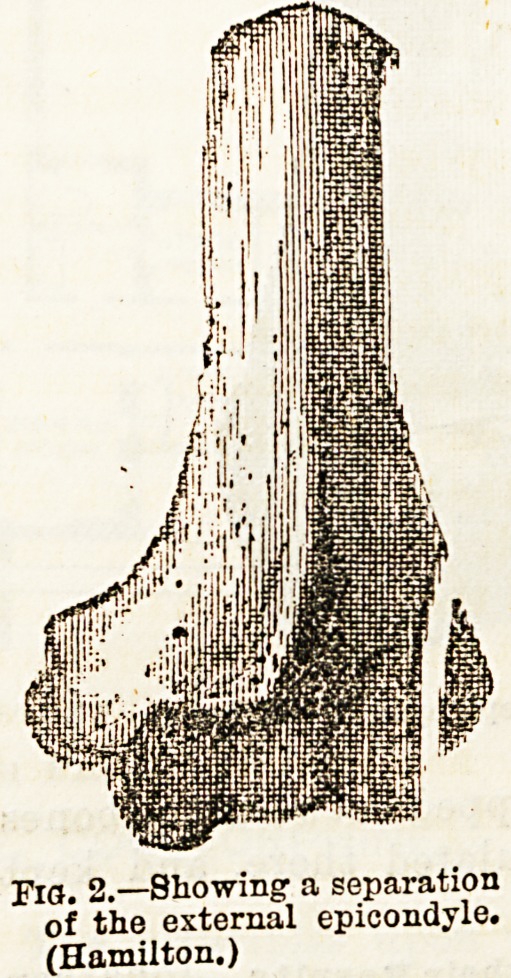


**Fig. 3. f2:**
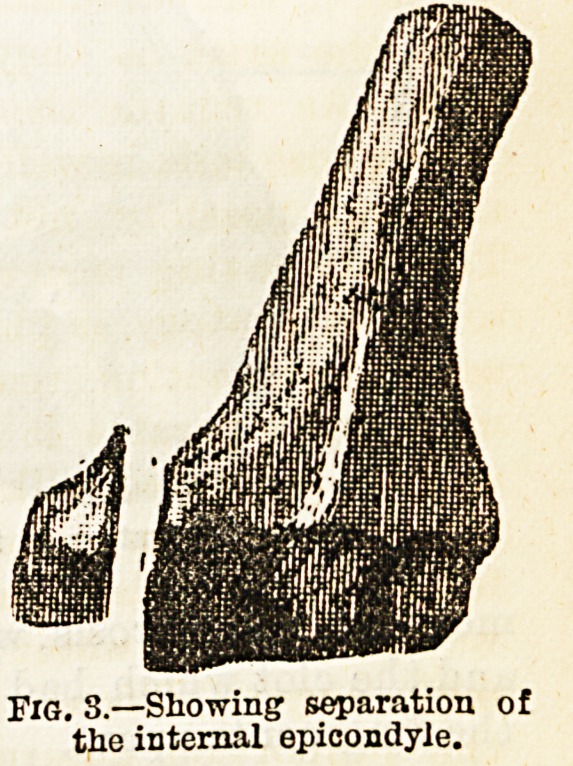


**Fig. 4. f3:**
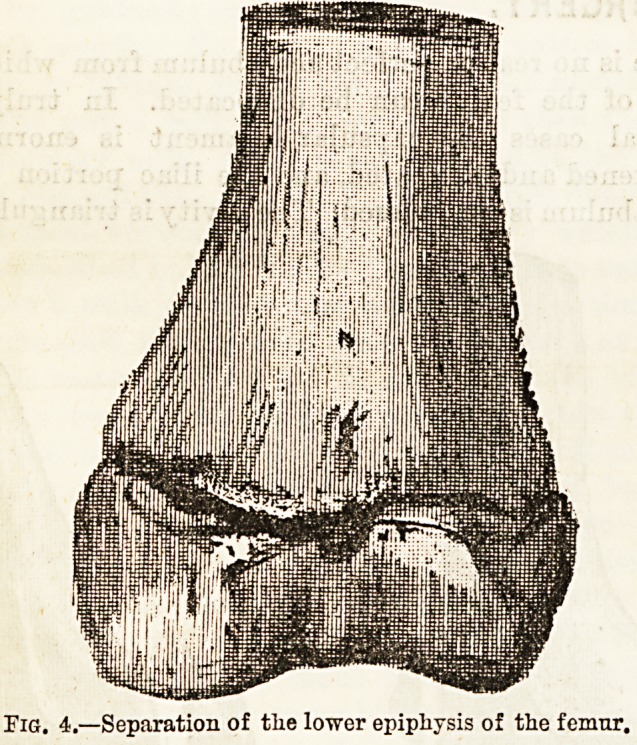


**Fig. 5. f4:**